# The Emerging Role of Oncolytic Virotherapy in Glioblastoma Management

**DOI:** 10.3390/cancers17213465

**Published:** 2025-10-28

**Authors:** Damir Nizamutdinov, Anna Sentmanat, Jing Tong, Xiaoming Qi, Yizhong Pan, Dan Qi, Erxi Wu, Jason H. Huang

**Affiliations:** 1 Department of Neurosurgery and Neuroscience Institute, Baylor Scott & White Health, Temple, TX 76508, USAjason.huang@bswhealth.org (J.H.H.); 2 College of Medicine, Texas A&M Health Science Center, Bryan, TX 77807, USA; 3 Department of Neurosurgery, The Fourth Hospital of Hebei Medical University, Shijiazhuang 050011, China; 4 Department of Neurology, Baylor Scott & White Health, Temple, TX 76508, USA; 5 Department of Neurosurgery, First Affiliated Hospital of Soochow University, Suzhou 215005, China; 6 Department of Neurosurgery, Baylor College of Medicine, Temple, TX 76508, USA; 7 Irma Lerma Rangel College of Pharmacy, Texas A&M University, College Station, TX 77843, USA; 8 LIVESTRONG Cancer Institutes, Department of Internal Medicine, Dell Medical School, The University of Texas at Austin, Austin, TX 78712, USA

**Keywords:** glioblastoma, solid tumor, oncolytic virotherapy, oncolytic viruses, tumor microenvironment, combination therapy, tumor treating fields, RANO 2.0 and iRANO criteria, immune checkpoint inhibitors

## Abstract

**Simple Summary:**

Glioblastoma is an aggressive brain cancer that almost always returns after surgery, radiation, and chemotherapy, and most patients live for little more than a year. This review explores an emerging option called oncolytic virotherapy using carefully engineered viruses that infect and destroy cancer cells while also alerting the immune system to attack the tumor. We describe why this strategy could succeed where current treatments fall short, compare leading viral platforms, and summarize recent findings from clinical studies, including combinations with radiation, chemotherapy, immune therapies, and even low-intensity electric field therapy. We also discuss the practical relevance of the blood–brain barrier, balancing safety and cost, and accurately measuring response. Our goal is to provide a clear map of progress, gaps, and next steps, enabling researchers and clinicians to design smarter trials and move more effective therapies toward patients.

**Abstract:**

Glioblastoma (GBM) is an aggressive and common form of central nervous system primary malignant tumor in adults. GBM accounts for about half of all gliomas. Despite maximal resection, radiotherapy, and temozolomide, median survival is still 12–15 months because of tumor heterogeneity, diffuse infiltration, and therapeutic resistance. Recurrence is nearly universal, underscoring the need for novel therapies. Oncolytic virotherapy demonstrates a promising strategy that combines direct tumor cell lysis with immune activation. Tumor-selective viruses replicate within malignant cells, induce cell death, and release tumor antigens, thereby reshaping the immunosuppressive microenvironment. Several viral backbones have advanced to clinical testing, including adenovirus (DNX-2401), herpes simplex virus (G47Δ, G207), poliovirus (PVS-RIPO), measles virus (MV-CEA), reovirus (pelareorep), vaccinia virus (Pexa-Vec), and vesicular stomatitis virus (VSV-GP). The approval of G47Δ in Japan for malignant glioma marks a milestone, with early trials demonstrating safety and signals of durable benefit, particularly in combination regimens. Current research emphasizes engineering viral genomes to enhance selectivity, immune stimulation, and resistance to clearance, while exploring synergistic combinations with radiotherapy, chemotherapy, immune checkpoint inhibitors, and tumor-treating fields. Advances in delivery, such as convection-enhanced infusion and blood–brain barrier modulation, are also under investigation. Despite obstacles, oncolytic virotherapy holds significant potential within multimodal GBM strategies.

## 1. Introduction

Glioblastoma (GBM) is the most common and aggressive primary brain cancer in adults [1]. It originates from glial stem cells (GSCs) or astrocytes that have lost normal regulatory function, resulting in high heterogeneity and complexity [2]. GBM is characterized by rapid proliferation, diffuse infiltration, and poorly defined margins with normal brain tissue [3], making effective treatment complicated.

Current classification systems recognize distinct molecular subtypes: mesenchymal, classical, and proneural, defined by characteristic mutations such as neurofibromatosis type 1 (NF1), phosphatase and tensin homolog (PTEN), and tumor protein 53 (TP53) in mesenchymal GBM; epidermal growth factor receptor (EGFR) amplification in classical GBM; and TP53, isocitrate dehydrogenase 1 (IDH1), and platelet-derived growth factor receptor alpha (PDGFRA) mutations in proneural tumors [2]. Due to intratumoral heterogeneity, multiple subtypes may coexist within the same lesion, complicating therapeutic targeting [4]. The field now relies on updated RANO 2.0 and iRANO response criteria rather than earlier Macdonald criteria to better assess pseudoprogression and immunotherapy-specific patterns in GBM trials [5]. Despite advances in multimodal therapy, the median survival of GBM patients remains only 12–15 months [4]. The Stupp regimen, which involves maximal safe surgical resection followed by radiotherapy and temozolomide (TMZ), remains the standard of care (Table 1) [4]. Surgery aims for gross total resection using modern intraoperative imaging and mapping (e.g., intraoperative ultrasound (ioUS)) or preoperative brain mapping (e.g., functional magnetic resonance imaging (fMRI)) [4]. However, infiltrative microscopic growth prevents complete excision, leading to recurrence within 6–9 months [2]. Carmustine wafers can be placed in resection cavities but confer only modest survival benefit (~2 months) [4]. The high recurrence rate and lack of efficacious second-line treatments contribute to the exceedingly poor prognosis for GBM patients [6].

Two FDA-approved chemotherapeutics are currently available: TMZ and bevacizumab [11]. TMZ, an alkylating agent introduced in 1999, induces DNA damage in tumor cells and is typically administered with radiotherapy [12]. Bevacizumab, a vascular endothelial growth factor (VEGF) inhibitor approved for recurrent GBM [13], restricts angiogenesis but provides limited survival improvement due to the blood–brain barrier (BBB) and active efflux mechanisms [14]. Moreover, the methylguanine-DNA methyltransferase (MGMT) DNA repair enzyme can reverse TMZ-induced alkylation, reducing efficacy in tumors with unmethylated MGMT promoters [2,15,16].

Radiotherapy, combined with TMZ, improves survival relative to radiation alone [2,17]. Modern precision techniques, such as intensity-modulated and helical tomotherapy, reduce collateral damage to normal tissue [12]. However, GBM’s hypoxic microenvironment limits radiotherapy effectiveness by promoting DNA repair and radioresistance [2,4,14]. Tumor Treating Fields (TTFields) is a non-invasive modality that applies low-intensity alternating electric fields to disrupt tumor cell mitosis and signaling pathways (e.g., VEGF, hypoxia-inducible factor (HIF), matrix metalloproteinases (MMPs)). TTFields are FDA-approved for newly diagnosed (with TMZ) and recurrent GBM but face cost and compliance barriers [4,16,18].

Immunotherapy, including immune checkpoint inhibitors (ICIs), chimeric antigen receptor T-cells (CAR-T), vaccines, and oncolytic virotherapy (OVT), represents the most recent therapeutic frontier [11,17,19]. Among these, OVT uniquely combines direct tumor lysis with immune activation [20]. Oncolytic viruses (OVs) are genetically engineered to selectively replicate in and destroy tumor cells while sparing normal tissue [21]. This dual mechanism, oncolysis plus in situ vaccination, amplifies anti-tumor immunity and may prevent recurrence [11,22].

The concept originated in 1991, when Martuza used a modified herpes simplex virus (HSV) against glioma [23]. Since then, several OVs have entered clinical trials with varying success [24]. G47Δ (teserpaturev, “Delytact”) became the first OV approved for malignant glioma in 2021 [25]. G207 (HSV-based) demonstrated safety and activity in Phase I/II trials [26]; DNX-2401 (adenovirus-based) achieved durable tumor control in select GBM patients [17]; and PVS-RIPO (poliovirus-based) induced robust immune responses with acceptable safety [27]. Newer “armed” viruses such as oHSV-IL-12 (M032) and DNX-2440 (adenovirus expressing OX40L) are being developed to enhance T-cell priming and overcome local immunosuppression [28,29].

Despite encouraging outcomes, OVT faces translational barriers, including limited intratumoral delivery and rapid antiviral clearance [27,30,31,32]. Ongoing studies aim to augment viral persistence and efficacy through combination with other immunotherapies [33,34]. This review summarizes recent progress and challenges in OVT, emphasizing its promise in GBM, one of the most treatment-resistant malignancies.

## 2. Types of Oncolytic Viruses, Mechanisms of Action, and Recent Clinical Advancements

OVT is a type of cancer treatment that uses genetically modified viruses to selectively infect and lyse targeted tumor cells, while remaining relatively harmless to normal cells [35]. OVs not only kill tumor cells through direct oncolysis but also release tumor antigens by inducing tumor cell lysis, thereby further activating the host’s immune system and forming a dual anti-tumor mechanism [36,37]. In recent years, multiple OVs have demonstrated therapeutic potential in various types of tumors in research and clinical trials [38,39]. For example, DNX-2401 (adenovirus), G207 (herpes simplex virus), and PVS-RIPO (poliovirus) have shown good safety and efficacy in clinical trials [40,41,42].

### 2.1. Adenovirus

Adenovirus is a double-stranded deoxyribonucleic acid (dsDNA) virus that lacks natural oncotropism [19]. To achieve tumor selectivity, adenoviruses are genetically engineered to replicate preferentially in cancer cells by deleting or modifying genes that are essential for replication in normal cells [30,43]. A classic example is the deletion of the E1B55K gene in early constructs such as ONYX-015, which enabled selective replication in p53-deficient tumors [30,43]. More recent clinically advanced vectors, such as DNX-2401 and CG0070, carry a 24-bp deletion within the E1A gene, which alters its binding to the retinoblastoma (Rb) protein. This modification restricts viral replication to tumor cells with defective Rb signaling, while sparing normal cells [31,43]. Importantly, the E1A gene itself is not completely deleted; instead, the mutation removes a short region responsible for binding Rb, thereby conferring tumor specificity while allowing E1A to be expressed and initiate replication.

In healthy cells, E2F transcription factors are tightly regulated by Rb. When Rb is mutated, deleted, or inactivated, E2F becomes constitutively active, driving uncontrolled cell-cycle progression. Adenoviral replication depends on this deregulated E2F activity, which the virus uses to amplify its genome and produce copies.

To further enhance tumor targeting, many adenoviral vectors incorporate an arginine-glycine-aspartic acid (RGD) fiber-knob modification that redirects viral attachment from the native coxsackievirus-adenovirus receptor (CAR) to αvβ3 and αvβ5 integrins [31,43]. These integrins are highly expressed on tumor cells and tumor-associated endothelial cells, particularly in invasive and angiogenic regions, providing an efficient and tumor-selective entry route. In some designs, natural adenoviral receptors are ablated and replaced with tumor-specific ligands, such as antibodies targeting human epidermal growth factor receptor 2 (HER2) or peptides recognizing EGFR [30].

Once inside the tumor cell, adenovirus expresses E1A and E1B early genes that drive the host cell into S phase, upregulate E2F, and promote viral DNA replication and cell lysis [43]. Thus, adenovirus serves as a genetically engineered oncolytic platform, with successive generations (e.g., ONYX-015, DNX-2401, CG0070) progressively improving tumor selectivity, safety, and clinical efficacy.

Clinical Advancements: DNX-2401 (tasadenoturev): a modified adenovirus that targets the integrin αvβ3, highly expressed on GBM cells. Recent clinical trials have demonstrated that DNX-2401 can significantly extend survival in some patients with recurrent GBM, in some cases surviving for more than three years [44]. Additionally, DNX-2401 is in a phase 1/2 study with pembrolizumab (CAPTIVE/KEYNOTE-192) [45]. DNX-2401 priming followed by PD-1 blockade yielded immune-inflamed microenvironments with preliminary evidence of clinical activity in a subset of patients [44,45]. Initial results suggest that this combination may further enhance anti-tumor immune response [46].

DNX-2440 (OX40L-armed): First-in-human data suggest the feasibility and early activity of this treatment in recurrent GBM [45].

### 2.2. Herpes Simplex Virus

Herpes Simplex Virus (HSV) is a dsDNA virus that has been modified to exhibit oncotropism [31]. Deletion of HSV virulence genes is done for both host safety and for tumor selectivity [47]. In particular, oncolytic HSV is modified to decrease expression of the gene responsible for ribonucleotide reductase (RR). Doing so enables the virus to be selective for tumor cells that are Rb-deficient and consequently overexpress E2F, which results in overproduction of tumor cell RR that HSV can use to replicate [48]. HSV can also be modified to delete infected cell protein 34.5 (ICP34.5), which is responsible for replication and the suppression of the type I interferons (IFN-1) pathway. Deletion of ICP34.5 enables selectivity for tumor cells, which are inherently deficient in components that generate an interferon alpha (IFN-α) response [48]. Capsid glycoproteins can also be modified to target tumor cells further [47]. ICP34.5, also known as γ34.5, is the major neurovirulence factor of HSV that counteracts the host’s protein kinase R (PKR)–mediated shutdown of protein synthesis and promotes viral replication in neurons. Its deletion prevents the virus from reversing the phosphorylation of eukaryotic translation initiation factor 2 alpha subunit (eIF2α), leading to reduced neurotropism and enabling selective replication in tumor cells with defective antiviral responses. This modification is essential for improving the safety profile of HSV-based OVs while maintaining their lytic activity in malignant tissues [48]. Clinical examples such as HSV1716, G207, and T-VEC (Talimogene laherparepvec) illustrate these rationally engineered HSV-based OVs, several of which have reached advanced clinical trials or regulatory approval.

Clinical Advancements: G207: a genetically modified HSV-1 OV with deletions of the ICP34.5 and ICP6 genes, enhancing its selectivity and safety. Recent clinical trials of Phase I/II have shown that G207 demonstrates a promising safety profile and preliminary efficacy in treating patients with recurrent GBM [49]. When combined with low-dose radiation therapy, G207 demonstrated enhanced therapeutic effects, highlighting the potential synergy between radiation and OVT [49].

In 2021, G47Δ (teserpaturev) became the first oncolytic HSV-1 therapy to gain approval in Japan for malignant glioma; ongoing postmarketing studies are assessing its long-term safety and survival outcomes in GBM [25].

T-VEC (Talimogene Laherparepvec): it was initially developed for melanoma, but its potential for treating GBM has also been explored recently. Combining T-VEC with other immunotherapies (such as ICIs) was the primary focus of its applications. Early results suggest that this combination therapy may improve survival outcomes in GBM patients [50]. Data in GBM remains limited; most experience is in melanoma. Combinatorial strategies with ICIs are under investigation but have not yet proven to be practice-changing for GBM [51].

M032 (oHSV-IL-12): Early human studies show acceptable safety; a pembrolizumab combination trial is enrolling [52,53].

### 2.3. Measles Virus

Measles Virus (MeV) is a negative-sense ribonucleic acid (RNA) virus that is oncotropic for CD46 expression on tumor cells [54]. MeV also uses signaling lymphocyte activation molecule (CD150/SLAM) and Nectin-4 as receptors, but CD46 is the most commonly overly expressed receptor on tumor cells [55]. As many tumor cells are deficient in generating innate antiviral immune responses, particularly in producing and responding to type I interferons (IFN-α/β). Since IFNs normally activate antiviral proteins that block viral replication and promote infected-cell apoptosis, their absence allows MeV to replicate more efficiently and spread within tumor tissue [54]. MeV can fuse infected cells into a syncytium. This results in the loss of membrane integrity and cell death through either apoptosis or autophagic mechanisms [55]. MeV can also induce a host immune response to the cells, leading to T cell infiltration and immune-mediated tumor destruction [54,55]. A clinical-grade carcinoembryonic antigen-expressing oncolytic MeV derivative (MV-CEA) has shown safety and early activity signals in recurrent GBM [56]. Both wild-type MeV and engineered derivatives such as MV-CEA, MV-NIS, and MV-GFP exhibit oncolytic potential, with the latter designed to enhance tumor selectivity and monitoring capability.

Clinical Advancements: MV-CEA: an oncolytic measles virus engineered to express the carcinoembryonic antigen (CEA), aiming to enhance anti-tumor immune responses [56]. A recent Phase I GBM study has demonstrated good safety and preliminary anti-tumor activity profiles for MV-CEA in recurrent GBM patients, with some showing anti-tumor activity [57].

### 2.4. Poliovirus

Poliovirus is a positive-sense RNA virus that can be oncotropic for cells expressing the poliovirus receptor (e.g., Necl-5, found in neuroectodermal cells) [58]. Polio is also highly restricted to target tissues and cells that express poliovirus receptor (CD155/PVR) [58]. Oncolytic poliovirus can be modified to replace the internal ribosome entry site (IRES) segment with genes from human rhinovirus 2 (HRV2), decreasing the pathogenicity of the virus without affecting its ability to replicate and destroy tumor cells [58]. Once a cell is infected, the production of viral 2A protease cleaves tumor cell elements involved in messenger RNA (mRNA) transport and protein synthesis, leading to cell death [58]. Wild-type poliovirus displays intrinsic oncotropism for CD155-expressing tumors, and engineered variants such as PVS-RIPO and PVS-HPV-16 leverage this natural property with improved safety and tumor selectivity.

Clinical Advancements: PVS-RIPO: an OV derived from the poliovirus, which targets tumor cells expressing the CD155 receptor. In recent Phase I trials, PVS-RIPO demonstrated safety and partial efficacy in patients with recurrent GBM. Several studies are investigating the potential of combining PVS-RIPO with ICIs to amplify its anti-tumor effects [59,60].

### 2.5. Reovirus

Reovirus is a dsRNA virus that preferentially replicates in cells with activated Ras signaling (often driven upstream by EGFR), resulting in protein kinase R (PKR) inhibition and permissive replication [61]. In tumor cells, EGFR can be overexpressed, leading to reticular activating system (RAS) activation and the inhibition of PKR, which generally functions to limit viral synthesis [61]. Thus, tumor cells have an increased susceptibility to reovirus, allowing them to be infected and subsequently lysed through apoptosis [61]. Reovirus is inherently oncolytic, with naturally occurring serotypes (e.g., Dearing Type 3 Reovirus, Reovirus T3D) exhibiting tumor selectivity that has been further optimized in clinical formulations such as Reolysin (Pelareorep).

Clinical Advancements: Pelareorep (Reolysin): a reovirus-based OV that targets tumor cells with active Ras signaling pathways [62]. Recent studies have focused on combining Reolysin with other strategies, such as radiation and chemotherapy, to enhance the potential efficacy of treating GBM. Early results indicate that Reolysin can enhance the anti-tumor effects of these therapies [63]. Clinical GBM data are mixed; synergy with chemoradiation is mechanistically plausible but not yet definitive.

### 2.6. Vaccinia Virus

Vaccinia virus (VAV) is a dsDNA virus in the Poxviridae family. It is modified to target tumor cells expressing tyrosine kinase (TK) [64]. The micropinocytosis is used by the virus to enter cells, where it replicates and causes lysis of the host cell [64]. Vaccinia can also be modified to target VEGF in tumor-associated endothelial cells, leading to disruption of the blood supply and vascular collapse of the tumor [64]. The VAV can also be armed with immunomodulatory genes to enhance antitumor immunity. The virus can produce chemokines and chemokine receptors to help improve its oncolytic abilities by reducing the clearance of the virus from the host [65]. Examples include Pexa-Vec (JX-594), GL-ONC1, and vvDD, which build on the virus’s replicative robustness while incorporating deletions or transgenes to enhance tumor specificity and immune activation.

Clinical Advancements: Pexa-Vec (JX-594): a modified vaccinia virus that induces robust immune responses by lysing tumor cells directly [66]. Pexa-Vec has shown promise in treating hepatocellular carcinoma (HCC). The development in HCC was halted after a negative phase III trial [67]. It is now being explored for the treatment of GBM; however, GBM exploration is limited and not currently a leading platform. Nevertheless, early clinical trials continue researching the safety and efficacy of using JX-594 in the treatment of GBM patients [63].

### 2.7. Vesicular Stomatitis Virus

Vesicular Stomatitis Virus (VSV) is a negative-sense RNA virus belonging to the Rhabdoviridae family. VSV uses low-density lipoprotein (LDL) receptors to enter cells. This results in broad tissue tropism [68]. Virus exhibits selectivity for cells lacking an antiviral IFN response, which makes it precise to target tumor cells in various tissue types [54]. The virus only replicates in the cytoplasm and can replicate rapidly, so only low doses of the virus are required to achieve an oncolytic effect [68,69]. VSV is naturally oncolytic in IFN-defective tumor cells, can be attenuated (e.g., VSV-IFNβ), and is being evaluated clinically as VSV-glycoprotein (GP)—(pseudotyped with lymphocytic choriomeningitis virus (LCMV) GP) and VSV-human Monocyte Chemoattractant Protein 3 (hMCP3) to reduce neurotropism and the ability to stimulate a host immune response against infected cells [68,69] (Table 2). Hence, VSV represents a naturally oncolytic virus refined through engineering for clinical translation.

Clinical Advancements: VSV-GP: a genetically modified vesicular stomatitis virus with reduced neurotoxicity and enhanced tumor selectivity. In preclinical GBM models, VSV-GP significantly inhibited tumor growth, and its efficacy was further enhanced when combined with ICIs [70]. Building on these preclinical findings, early-phase clinical trials across solid tumors, including brain cancers, are currently evaluating its safety and preliminary therapeutic activity in GBM patients [71] (Table 3). Human data remain limited but will be critical to confirm the translational potential observed in experimental models.

Table 2 outlines the main types of OVs, their characteristics, areas of application, and representative examples, providing an overview of their potential in cancer treatment.

Table 3 below outlines key characteristics, clinical status, advantages, and limitations of leading OV platforms evaluated for the treatment of GBM.

## 3. Combination Therapies of Oncolytic Virotherapy

### 3.1. Combination with Immune Checkpoint Inhibitors

ICIs, such as PD-1/PD-L1 and cytotoxic T-lymphocyte-associated protein 4 (CTLA-4) inhibitors, can enhance the immune response of the host against tumors by blocking immune checkpoint signaling and deregulating T cells [51]. However, GBM has a more limited response to ICIs, in part due to its highly immunosuppressive microenvironment and low immunogenicity [72,73]. By directly targeting and lysing tumor cells, large amounts of tumor antigens are released to the extracellular matrix. Thus, OVs can activate tumor-specific T cells and effectively induce a strong local inflammatory response (upregulate antigen presentation). OVs can disrupt the immunosuppressive state of the tumor microenvironment (TME) and reveal the benefits of ICI [74]. These mechanisms of action have a synergistic effect with the action of ICIs, so that the combination therapy of OVs and ICIs can significantly enhance the immune response against GBM and improve the therapeutic outcomes [75].

### 3.2. Combination with Radiotherapy

Radiation therapy (RT) is a standard treatment for GBM, which inhibits tumor growth by damaging the DNA of tumor cells and inducing cell death. Studies have shown that RT can increase the susceptibility of tumor cells to OV infection, in part because RT can cause a DNA damage response, alter the TME, and promote viral replication and spread [76,77]. Additionally, RT increases the release of tumor antigens and enhances the effectiveness of antigen presentation, thereby expanding the immune response of the host [66]. The combination of OVT and RT can further enhance this effect by inducing tumor cell lysis and simultaneously activating the immune system, resulting in an enhanced antitumor effect [78].

### 3.3. Combination with Targeted Therapy

Targeted therapies inhibit the spread and tumor growth by targeting unique molecules or cellular signaling cascades in tumor cell biology. GBMs are known to have complex molecular profiles and diverse mutations, making targeted therapies a viable option. The combination of OVs and targeted therapies can achieve synergistic effects through multiple mechanisms. Some targeted agents can inhibit the antiviral response of tumor cells, thereby enhancing the infectivity of OVs [79,80]. In addition, targeted therapies can also improve the efficacy of OVs by altering the biological properties of tumor cells (e.g., by increasing the expression of viral receptors or inhibiting antiviral signaling pathways) [73].

### 3.4. Combination with Chemotherapy

Chemotherapy is effective in killing rapidly proliferating tumor cells by interfering with the DNA replication and cell division nature of cancer cells. However, chemotherapy has downsides like a lack of specificity, being toxic to healthy cells, and causing immunosuppression. The combination of OVT and chemotherapy can address these shortcomings. Chemotherapy can weaken the immunosuppressive TME shield and therefore increase the efficiency of viral infection and tumor lysis [81]. Additionally, specific chemotherapeutic agents can induce immune cell death (ICD), release tumor antigens, and stimulate immune activation [82]. This synergistic effect can enhance the antitumor effect and reduce the risk of tumor recurrence through multiple pathways [83]. For example, HSV infectivity and replication are increased when it is paired with TMZ [47,50]. Gemcitabine was seen to have a similar effect on the infectivity of reovirus and vaccinia when used in combination [61].

### 3.5. Combination with TTFields

While TTFields are still a relatively novel treatment for GBM, they have been shown to modify the TME and increase the permeability of the BBB [84]. Used in conjunction with OVs, TTFields could enhance viral penetration and help trigger a more robust immune response [85,86]. As both of these treatments are noninvasive, combination therapy could be both safer and more effective for the host.

### 3.6. Combination with Other Immunotherapies

In addition to ICIs, OVs can be used in combination with other immunotherapies, such as CAR-T therapy and vaccine therapy [14,87]. These immunotherapies activate and enhance the host immune response through various mechanisms. OVs can achieve more potent antitumor effects by inducing the release of tumor antigens and local inflammatory cytokines and chemokines, increasing the penetration and killing ability of CAR-T, and/or enhancing vaccine-directed specific T-cell responses [88]. One example of this can be seen in the genetic modifications of vaccinia that enable the OV to express T cell costimulatory molecules, thereby enhancing the tumor-directed killing capacity of CAR-T therapy [64]. Additionally, immunotherapies can increase the efficacy of OV replication by further suppressing the antiviral IFN pathway in the TME [69,74].

## 4. Challenges and Limitations of Oncolytic Virotherapy

OVTs offer considerable promise in the treatment of GBM; nevertheless, their clinical implementation continues to encounter several obstacles:

### 4.1. Viral Delivery and Distribution

Heterogeneity and the BBB remain central obstacles. Convection-Enhanced Delivery (CED), repeat dosing, or pairing with BBB-opening technologies (such as focused ultrasound (FUS) or osmotic) are being explored to overcome these challenges [89,90,91,92]. Not all of the OVs have natural oncotropism; genetic engineering is needed to help some of them target tumor cells effectively [19]. One concern is regarding the stability of the viral genome when engineering without undergoing genetic mutations. RNA viruses are less stable and can affect the ability of RNA OVs to target tumors without causing more widespread damage [61]. Considerable heterogeneity of GBM and the intricate TME, particularly the presence of the BBB, may also impede the virus’s capacity to disseminate efficiently and target the tumor region, rendering it challenging for the virus to be uniformly distributed within the target structure in the brain [34,93].

### 4.2. Host Immunity and Resistance

Some of the viruses used, such as poliovirus and measles virus, face the considerable challenge of pre-existing host immunity due to widespread vaccination. Other viruses, such as adenovirus, may face the same issues due to pre-existing infection [31,94]. As a result, the body’s immune system may rapidly identify and eliminate the injected virus, which significantly reduces the duration and efficacy of the virus in Tumor cells [21,32]. Using viruses as a recurrent therapy may also induce host resistance to them through the formation of antibodies or other neutralizing mechanisms [31,61]. Therefore, with the continuation of treatment, tumor cells may gradually develop resistance to the virus, which may limit the long-term efficacy [16]. As a result of this potential neutralizing mechanism, the development of multiple viruses with distinct mechanisms of action or combination strategies with other therapeutic modalities may prove instrumental to overcome this limitation [34]. To address this obstacle, researchers are exploring ways to enhance the virus’s immune escape ability through genetic engineering techniques or to combine the use of immunosuppressants to prolong the time the virus remains in the body [74,95].

### 4.3. Side Effects

The safety and potential adverse effects of the virus must be taken into account, especially since some of the viruses are highly pathogenic. Inadequate lowering of viral pathogenicity can lead to systemic toxicity, particularly in the RNA viruses that are difficult to engineer [54]. Care must also be exercised with some of the DNA viruses, especially HSV, because HSV can remain latent in the host and potentially cause long-term side effects, particularly in immunocompromised individuals [28]. With many positive safety reports observed in clinical trials using OVs, they are still prone to some unexpected immune responses or other adverse reactions, particularly at higher doses or in treatments with multiple injections [73]. The use of OVs in combination with other treatment modalities can also maximize the damage to non-tumor cells. Inappropriate dosing or combination use can lead to severe immunosuppression and toxicity, as one study with systemic reovirus therapy has reported [96]. Further research is required to optimize the virus design and the administration regimen to maximize therapeutic efficacy while minimizing adverse effects [97].

To overcome these challenges, some of the platform-specific risks can be avoided using the approaches below: HSV latency risk can be mitigated by γ34.5 (ICP34.5) and UL39 (ICP6) deletions that blunt neurovirulence, and by retaining HSV-thymidine kinase (TK) to preserve acyclovir/ganciclovir sensitivity as a pharmacologic “off” switch [98,99]. VSV-related neurotropism/neurovirulence can be attenuated by arming with IFN-β (VSV-IFNβ) to exploit intact antiviral signaling in normal tissue, or by pseudotyping (e.g., VSV-GP) to reduce neuronal targeting; additional detargeting strategies may be layered as needed [100].

### 4.4. Efficacy and Cost

Because it is novel, virotherapy is very expensive, especially when considering that it is not the first-line treatment for GBM [101]. The cost of the therapy alone hinders its full potential. Virotherapy is also not recommended as monotherapy, due to uncertainty about its ability to generate complete regression of tumors [61]. As such, virotherapy must be used in conjunction with another of the already established treatment regimens for GBM, further increasing the cost and timeframe of successful treatment [61].

## 5. Discussion

GBM remains the most challenging malignancy in neuro-oncology, with aggressive infiltration and resistance to standard therapies, and limited long-term survival despite surgery, radiotherapy, and chemotherapy [2,14,102]. While immunotherapies have transformed care in other cancers, efficacy in GBM has been modest [103,104]. OVT represents an emerging and biologically distinct approach capable of directly lysing tumor cells while simultaneously activating systemic antitumor immunity [105]. This dual mechanism distinguishes OVT from other immunotherapies and supports its integration with established GBM treatments. The following sections synthesize platform traits, selectivity, therapeutic positioning, delivery, safety, and future directions.

### 5.1. Types of Viruses

A variety of viral backbones are currently under investigation for OVT in GBM. Their biological diversity, ranging from DNA to RNA genomes, offers different balances of stability, pathogenicity, and engineering potential. Adenoviruses are relatively low in pathogenicity, highly stable, and possess large double-stranded DNA genomes that permit multiple gene insertions and receptor modifications [30,43,61,64,65]. HSV-based vectors similarly leverage large genomes but require deletions of neurovirulence genes to ensure safety [47]. By contrast, RNA viruses such as reovirus, measles, and poliovirus provide strong intrinsic immunogenicity and natural tumor selectivity. However, their genetic instability and neurotropism require modification to make it less harmful [54,55,58,69]. VSV combines high replication efficiency with innate selectivity for interferon-defective cells, but also requires pseudotyping or IFN-β arming to reduce neurotoxicity. Viral pathogenicity, pre-existing immunity, and payload flexibility are therefore major determinants in platform selection [106].

Overall, DNA viruses offer customizable stability and large genetic capacity, whereas RNA viruses provide potent immunogenicity but demand tighter safety controls. The ideal choice depends on the therapeutic goal and intended combination partners.

### 5.2. Specificity of Viruses

The success of OVT ultimately relies on tumor selectivity. Naturally OVs, such as VSV, reovirus, measles, and poliovirus, exploit defects in antiviral signaling (e.g., PKR or interferon pathways) to replicate preferentially in tumor cells [19,64]. This intrinsic selectivity simplifies vector design and may reduce manufacturing costs. However, natural tropism rarely ensures safety, and attenuation remains essential, particularly for neurotropic viruses [59,70]. In contrast, engineered viral vectors (e.g., adenovirus, HSV) achieve precision through targeted deletions and promoter control. Tumor-specific promoters, microRNA targets, or virulence gene deletions can restrict replication to malignant cells. At the same time, the use of immunostimulatory transgenes such as Granulocyte-Macrophage Colony-Stimulating Factor (GM-CSF) or Interleukin-12 (IL-12) enhances immune priming [106,107]. Such modifications also help overcome pre-existing immunity by altering antigenicity [29].

In GBM, minimizing off-target neurotoxicity and overcoming immune evasion are paramount. Engineered vectors, by allowing precise control of replication, payload delivery, and immune activation, remain the most versatile tools for future clinical development.

### 5.3. Monotherapy Versus Combination Approaches

Although durable responses have occasionally been observed with single-agent virotherapy [108], these remain uncommon. Trials with DNX-2401 reported long-term survivors [44], and the approval of oHSV G47Δ (teserpaturev) confirmed that virotherapy can achieve clinical recognition [25]. Yet, across studies, most patients benefit only when OVs are integrated with other modalities.

The limited success of monotherapy reflects several biological barriers: immune-mediated viral clearance, restricted intratumoral distribution, and heterogeneous receptor expression. In contrast, combination strategies, particularly with radiotherapy, chemotherapy, or immune checkpoint blockade, consistently demonstrate enhanced efficacy by broadening both immune and cytotoxic mechanisms [45,109]. These findings position OVT as a potent adjuvant or sensitizer within multimodal regimens rather than a stand-alone treatment.

### 5.4. Integration with Chemotherapy, Radiotherapy, and Immunotherapy

Radiotherapy enhances OVT efficacy by inducing DNA damage, promoting viral replication, and releasing tumor antigens that heighten immune recognition [54,61,110]. The clinical effectiveness of G207 plus low-dose radiation exemplifies this translational success [109]. Chemotherapy interactions are more complex. Agents such as TMZ can improve HSV infectivity and reovirus replication, while immunosuppression from TMZ may counterbalance immune activation [111]. Optimizing sequencing and dosing remains crucial. Synergistic activity has also been noted with gemcitabine in preclinical reovirus and vaccinia models [112]. Evidence from animal studies further supports the potential of combining OVT with chemotherapy and radiation, and even with surgical resection, in achieving prolonged survival [50,113,114].

The most transformative results arise when OVT is paired with immunotherapies. Infection-induced inflammation can amplify immune activation and checkpoint responsiveness [14,115,116]. OVs can convert GBM’s “cold” TME into an inflamed, antigen-rich niche through immunogenic cell death, antigen release, and cytokine upregulation [117,118,119,120]. This renders tumors more responsive to ICIs, as demonstrated in DNX-2401 plus pembrolizumab, which promoted cytotoxic T-lymphocyte (CD8+) infiltration and PD-L1 expression [44,45]. Similarly, oHSV-IL-12 vectors enhance T-cell recruitment and cytokine production [121,122,123]. While cost and individualized vector design remain limiting factors [101,124], these multimodal strategies represent the most promising path forward for GBM (Table 4).

### 5.5. Current Therapies Challenges, and Rationale for Virotherapy

The Stupp regimen, combining maximal safe resection, radiotherapy, and TMZ, has remained the standard of care for nearly two decades, yet median overall survival rarely exceeds 15 months [11,12]. Despite its widespread use, this approach faces intrinsic biological barriers. GBM exhibits extensive intratumoral heterogeneity, encompassing distinct mutational subclones, metabolic adaptations, and TME interactions that enable treatment-resistant populations to persist [14]. Radiotherapy efficacy is further compromised by hypoxia, which limits DNA damage fixation, whereas TMZ effectiveness diminishes in tumors with unmethylated MGMT promoters that restore DNA repair capacity [14]. Even newer modalities, such as TTFields, have been limited by compliance and cost, while ICIs have faced barriers due to GBM’s profoundly immunosuppressive TME [2,86].

OVT offers a unique opportunity to overcome several of these limitations simultaneously. By exploiting tumor-specific mutations for selective replication, OVs can bypass conventional resistance pathways, induce immunogenic cell death, and stimulate systemic antitumor immunity [29]. Nonetheless, significant challenges remain in translating these mechanisms into consistent clinical benefit. Achieving uniform viral distribution within the tumor mass, preventing rapid immune clearance, and extending intratumoral persistence are ongoing hurdles [125,126,127,128]. The BBB further restricts delivery, necessitating innovative approaches such as convection-enhanced delivery or direct intratumoral injection. Parallel strategies, including transient immunosuppression and genetic engineering to enhance viral stealth and safety, seek to prolong therapeutic activity while minimizing adverse reactions. Lessons from negative trials, such as Toca 511 + 5-FC in recurrent high-grade glioma, emphasize the importance of biomarker-guided patient selection and rigorous, well-controlled clinical designs [129,130].

### 5.6. Comparative Analysis of Viral Platforms

Each viral platform presents distinct advantages and limitations. HSV derivatives (G47Δ, G207) exhibit high payload capacity, neuronal tropism compatible with glial tumors, and a favorable safety record after neurovirulence gene deletions [25]. Adenoviruses (DNX-2401) combine genetic stability with efficient production and manageable immunogenicity but require integrin-targeted modifications to enhance tumor entry [45].

Poliovirus-based OVs (PVS-RIPO) leverage CD155 expression and have produced extended survival in subsets of GBM patients [29,59]. Measles virus derivatives (MV-CEA, MV-NIS) target CD46 with high selectivity and enable real-time imaging of viral replication, though pre-existing immunity can reduce efficacy [54,56]. Reovirus (Pelareorep) harnesses Ras pathway activation common in GBM, but neutralizing antibodies can limit systemic use [62]. Vaccinia virus (Pexa-Vec, vvDD, GL-ONC1) and VSV are adaptable backbones with robust immunogenicity [64,69]. While vaccinia development slowed after HCC trial setbacks [131], VSV pseudotyping and IFN-β arming have produced potent yet safer vectors [70].

Collectively, these data indicate that HSV and adenovirus remain the most clinically advanced platforms, while poliovirus and measles exploit natural tropisms, and vaccinia and VSV serve as promising chassis for next-generation engineering [29].

### 5.7. Delivery Challenges and the Blood–Brain Barrier

Effective intratumoral delivery remains a defining challenge in OVT. The BBB limits systemic access, and most current trials rely on direct injection or CED during tumor resection to distribute the virus into peritumoral zones [29,132]. Despite high local concentrations, these methods are invasive and complex to repeat.

Emerging strategies aim to improve repeatable, minimally invasive delivery. MRI-guided FUS with microbubbles can transiently open the BBB, permitting viral entry without surgery, and has demonstrated early safety in recurrent GBM [91]. TTFields may also enhance BBB permeability [133,134]. Intra-arterial infusion with osmotic agents such as mannitol has shown feasibility, although procedural risks remain [135,136]. Systemic delivery innovations, including nanocarriers and exosome-based transport, are being investigated to complement local approaches. Integrating these technologies may ultimately enable sustained, multi-dose OVT regimens.

### 5.8. Safety, Cost, and Regulatory Perspectives

Safety considerations continue to shape OVT design. For DNA viruses such as HSV, deleting ICP34.5 and UL39 genes mitigates neurovirulence while preserving a pharmacologic “off switch” via retained thymidine kinase [98,99,137], for RNA viruses like VSV and poliovirus, attenuation (e.g., VSV-IFNβ, pseudotyping) limits systemic toxicity [100,131].

Economic and regulatory challenges are intertwined. Manufacturing OVs requires high-level biocontainment, individualized dosing, and often neurosurgical delivery, all of which increase costs [29,138]. Nonetheless, the conditional approval of G47Δ demonstrates that consistent safety and efficacy data can yield regulatory success [25]. Harmonized trial design and global standards will be essential to expand access.

### 5.9. Future Directions

Next-generation OVs are being designed to act as multifunctional immunomodulators, expressing IL-12, GM-CSF, or ICIs directly within the TME [39]. Advances in synthetic biology now enable personalized virus design based on tumor receptor profiles, patient immunity, and antiviral gene expression [29].

Combining OVs with BBB-modulating technologies (FUS), cell-based immunotherapies (CAR-T, dendritic-cell vaccines), or oncolytic-nanoparticle hybrids could yield highly targeted, repeatable treatment strategies [29]. Artificial intelligence and imaging biomarkers may assist in real-time monitoring of viral spread and immune activation. The adoption of the RANO 2.0 and iRANO frameworks will be vital for consistently evaluating responses and distinguishing pseudo-progression from true recurrence [5].

With these evolving directions, OVT stands poised to transform GBM therapy by bridging precision virology, immuno-oncology, and neuro-oncology into an integrated therapeutic frontier.

## 6. Conclusions

OVT has progressed from a theoretical concept to a clinically validated therapeutic strategy, with early approvals such as G47Δ demonstrating its translational potential. Its most significant value in GBM lies not in isolation but as part of multimodal regimens that integrate direct oncolysis with immune activation and synergy with established therapies. While challenges remain in optimizing viral specificity, delivery, and durability of response, ongoing advances in genetic engineering and clinical trial design continue to push the field forward. Collaboration of virologists, immunologists, and neuro-oncologists will be essential to translate these advances into tangible benefits for patients. With continued innovation, OVT holds the potential to transform the treatment of GBM, offering meaningful improvements in survival and quality of life where effective options remain limited.

## Figures and Tables

**Table 1:** Current Therapeutic Modalities for GBM: Mechanisms, Benefits, Limitations, and Clinical Outcomes.

Therapy	Mechanism	Benefits	Limitations	Efficacy	Examples	Combination
Surgery	Gross resection of the tumor	Quicker, no systemic side effects, potential for complete resection	Inability to remove microscopic tumor growth leads to recurrence	Up to 61% increased 1-year survival with gross total resection [7]	Gross total resection, Subtotal resection	Can be used with chemotherapy and radiotherapy
Chemo therapy	Alkylation and damage of tumor cell DNA, sensitization of tumor cells to radiation	Directly cytotoxic, can be combined with radiation for increased efficacy	Cancer cell resistance through DNA repair genes and efflux pumps	Up to 2.5 months of increased survival when used with RT [8]	TMZ, Bevacizumab carmustine	Can be used after surgery, augments the efficacy of radiation
Radiation	Use radioactive particles to cause oxidative damage to tumor cells	Can spare healthy tissue, directly cytotoxic	A hypoxic tumor environment renders it less effective	Up to 2.5 months of increased survival when used with CT [8]	X-ray photons, gamma photons	Increased efficacy with chemotherapy
TTFields	Uses frequency and electric fields to interfere with tumor cell replication and growth	Noninvasive	Expensive	Up to 4 months increased overall survival when used with TMZ [9]	N/A	Can be used with TMZ
Immuno therapy	Therapies that can modulate/enhance host immune responses to tumors	Can modulate the tumor microenvironment and induce host immune response	Novel	Up to 6 months increased survival rate when combined with the Stupp regimen [10]	CAR-T cells, Immune checkpoint inhibitors, vaccines, and oncolytic viruses	Novel, but can be used with the Stupp regimen

This table provides an overview of established and emerging therapeutic strategies for GBM, summarizing their mechanisms of action, advantages, limitations, survival benefits, representative examples, and potential for use in combination regimens. Chimeric antigen receptor T-cell (CAR-T); tumor treating fields (TTFields); temozolomide (TMZ); deoxyribonucleic acid (DNA), not available (N/A).

**Table 2:** Main Types of Oncolytic Viruses, Their Characteristics, Applications, and Examples.

Virus Type	Genetic Material	Mechanism of Action	Applications	Examples
Adenovirus	dsDNA	Engineered with E1B55K or E1A gene deletions that permit replication only in tumor cells lacking functional p53 and Rb pathways, thereby sparing normal cells	Solid tumors	DNX-2401 [17,40,41,42], ONYX-015 [30,43], CG0070 [31,43]
Herpes Simplex Virus	dsDNA	Attenuated by deletion of neurovirulence gene γ34.5 and ICP6 (ribonucleotide reductase) so replication occurs selectively in Rb-pathway-deficient tumor cells	Solid tumors	G207 [26,49], T-VEC (Talimogene laherparepvec) [50,51], HSV1716 [47,48], R7020 [47,48]
Measles Virus	(−) RNA	Naturally targets cells expressing CD46 and signaling lymphocyte activation molecule (SLAM/CD150), both highly expressed on many tumor cells; oncolysis occurs via syncytia formation and immune activation.	Hematologic and solid tumors	MV-CEA [56,57], MV-NIS [54,55,56], MV-GFP [54,55,56]
Poliovirus	(+) RNA	Exploits overexpression of CD155 (poliovirus receptor) in malignant cells; recombinant strains such as PVS-RIPO are engineered for safety with attenuated neurovirulence.	Solid tumors	PVS-RIPO [27,58,59,60], PVS-HPV-16 [58]
Reovirus	dsRNA	Naturally replicates in cells with activated Ras or EGFR signaling pathways that inhibit the antiviral protein kinase R (PKR), enabling selective oncolysis.	Solid tumors, multiple myeloma	Reolysin (Pelareorep) [62,63], Dearing Type 3 Reovirus [61] Reovirus T3D [61]
Vaccinia Virus	dsDNA	Modified by deletion of thymidine kinase (TK) and vaccinia growth factor (VGF) genes; relies on high TK expression and EGF signaling found in cancer cells	Solid tumors	Pexa-Vec (JX-594) [63,66,67], GL-ONC1 [64,65], vvDD [64,65]
Vesicular Stomatitis Virus	(−) RNA	Naturally infects cells with impaired type I interferon (IFN) response; oncolytic variants (e.g., VSV-GP, VSV-IFNβ) show enhanced tumor selectivity and immune stimulation.	Hematologic and solid tumors	VSV-GP [70,71], VSV-IFNβ [68,69], VSV-hMCP3 [68,69]

The table summarizes representative OVs currently studied in oncology, highlighting their biological properties, therapeutic applications, and notable examples to illustrate their potential in cancer treatment. Both naturally occurring and genetically engineered oncolytic viruses are included, reflecting the diversity of mechanisms and design strategies used in current research.

**Table 3:** Comparison of Oncolytic Virus Platforms Applied to GBM Therapy.

OV Platform (Examples)	Key Entry/Selectivity	Engineering/Payload	Typical GBM Delivery	Clinical Status & Notable Combination Data	Strengths	Watch-Outs
**Adenovirus (DNX-2401, DNX-2440-OX40L)**	RGD-modified fiber targets αvβ3/αvβ5; E1A 24 bp deletion → Rb-defect selectivity	Moderate payload; OX40L and other immunomodulators possible	Intratumoral (stereotactic); peri-cavity dosing	DNX-2401 + pembrolizumab (KEYNOTE-192) inflames GBM; DNX-2440 (OX40L) early feasibility	Well-characterized; synergistic with PD-1 blockade	Pre-existing anti-Ad immunity; invasive delivery
**HSV-1 (G207, G47Δ/teserpaturev, M032)**	Nectin-1/HVEM via gD; selectivity via ICP34.5/ICP6 deletions	Large DNA genome; high transgene capacity (e.g., IL-12)	Intratumoral/peri-resection cavity; convection-enhanced delivery (CED)	G47Δ (Japan, approved 2021); G207 safe with RT synergy; M032 (IL-12) + pembrolizumab ongoing	Arming-friendly; regulatory precedent in GBM	Requires local delivery; theoretical latency issues mitigated by deletions
**Poliovirus (PVS-RIPO)**	Targets CD155 (Necl-5), highly expressed in GBM	RNA virus; HRV IRES replacement for attenuation	Intratumoral infusion (CED)	Phase I: safety with OS plateau; combinations with ICIs under study	Natural neurotropism to GBM; durable-response tail in subset	Pre-existing immunity; catheter-based delivery
**Measles (MV-CEA)**	CD46 (overexpressed on tumors); also CD150, nectin-4	RNA virus; CEA reporter allows noninvasive monitoring	Intratumoral/peri-resection cavity	Phase I in recurrent GBM: safe with preliminary activity	Trackable via serum CEA; strong preclinical GBM data	Neutralized by measles immunity; RNA stability
**Reovirus (Pelareorep/Reolysin)**	Replicates in RAS-activated/PKR-impaired cells (often EGFR-driven)	RNA virus; small payload; IV delivery feasible	Intratumoral or systemic	Mixed GBM outcomes; synergy with chemo/RT preclinically	Systemic potential; well-studied biology	Neutralizing antibodies are common; inconsistent GBM efficacy
**Vaccinia (Pexa-Vec/JX-594)**	Broad tropism; TK-deletion confers tumor selectivity	Large DNA genome; payload capacity for cytokines, VEGF inhibitors	Intratumoral or systemic	GBM exploration limited; HCC phase III negative	High payload; strong innate immune activation	Program setbacks in HCC; GBM data sparse
**VSV/VSV-GP**	LDL receptor entry; tumor selectivity in IFN-deficient cells	RNA virus; attenuated forms (e.g., VSV-IFNβ, VSV-GP)	Intratumoral or systemic (early trials)	First-in-human VSV-GP: feasible; GBM data preliminary; ICI combos in testing	Potent oncolysis; ICI synergy rationale	Neurotoxicity risk (mitigated by pseudotyping/IFNβ insertion)

**Table 4:** Advantages and Disadvantages of Combination Therapy in GBM.

Advantages of Combination Therapy	Disadvantages of Combination Therapy
Change the activity of the TME (“Cold” to “Hot”)	Additive toxicity and immunosuppression risks
Multi-axis killing (Oncolysis + RT/Chemo + ICI)	Complex logistics and higher cost
Potential BBB/TME modulation (TTFields, FUS)	Interpretive challenges with imaging (pseudoprogression diagnosis with use of iRANO vs. RANO 2.0)

This table summarizes the potential benefits and limitations of multimodal therapeutic strategies in glioblastoma (GBM), including immunological conversion of the tumor microenvironment (TME), synergistic tumor-killing mechanisms, and modulation of the blood–brain barrier (BBB) and TME.

## Data Availability

No new data were created or analyzed in this study.
